# Individual Basis for Collective Behaviour in the Termite, *Cornitermes cumulans*

**DOI:** 10.1673/031.008.2201

**Published:** 2008-03-18

**Authors:** Octavio Miramontes, Og DeSouza

**Affiliations:** ^1^ Departamento de Sistemas Complejos, Institute de Fisica, Universidad Nacional Autónoma de México (UNAM), Cd Universitaria 04510 DF, Mexico; ^2^ Departamento de Biologia Animal, Universidade Federal de Viçosa, 36.570-000 Viçosa, MG, Brazil

**Keywords:** self-organisation, pattern emergence, sociality, insects, agent based models, complex systems

## Abstract

Interactions among individuals in social groups lead to the emergence of collective behaviour at large scales by means of multiplicative non-linear effects. Group foraging, nest building and task allocation are just some well-known examples present in social insects. However the precise mechanisms at the individual level that trigger and amplify social phenomena are not fully understood. Here we show evidence of complex dynamics in groups of the termite, *Cornitermes cumulans* (Kollar) (Isoptera: Termitidae), of different sizes and qualitatively compare the behaviour observed with that exhibited by agent-based computer models. It is then concluded that certain aspects of social behaviour in insects have a universal basis common to interconnected systems and that this may be useful for understanding the temporal dynamics of systems displaying social behaviour in general.

## Introduction

Is there a minimal number of individuals necessary for a given trait of social behaviour to appear? Why do individuals present apparently chaotic and unpredictable activity, while the groups they belong to present ordered patterns? How do interactions turn uncorrelated and disordered individual behaviour into more ordered and coherent collective behaviour? Why does temporal activity in social groups seem to have self-similar patterns? In recent years, there is a renewed interest for addressing these kind of questions coming out from a more basic yet unanswered problem in evolutionary biology: how does collective social behaviour emerge out of cooperating individuals interacting locally?

While these questions are general for all social organisms, termites present a particular set of well known behaviours, ranging from very simple self-grooming to extremely elaborate collective nest building. How such behaviours are triggered and controlled is not yet fully understood, but there is growing evidence that common general principles are in action: termite collective patterns emerge spontaneously from the concurrent action of simple individuals, performing simple local tasks with no information whatsoever on the global pattern to be achieved. When an insect colony is viewed as a complex sytem, “[...] common principles exist at the organismic and superorganismic levels, thus between individual insects and the tightly integrated colonies they compose” (Wilson & Holldobler 2005).

Several previous works have pointed to the emergence of collective behaviours in several organisms including termites. Recent examples range from behaviour amplification and mass recruitment in ants (Jeanson et al. 2004; Dussutour et al. 2005), self-organised temporal synchronisation in ants (Miramontes et al. 2001), colony-size influence on individual performance in wasps (Karsai et al. 1998), nest construction in spiders (Bourjot et al. 2003), self-organised aggregation in cockroaches (Jeanson et al. 2005), synchronization in fireflies (Camazine et al., 2001), among many others. In termites there are a number of complex behaviours that have been studied using agent-based models. Examples include self-organised nest construction (Deneubourg 1977; Courtois et al. 1991; Bonabeau et al. 1997 ; O'Toole et al. 1999, 2003), social facilitated survival (Miramontes and DeSouza 1996), disease transmission (Pie et al. 2004) and individual recognition (Copren et al. 2005).

Agent-based models have proved to be powerful tools for exploring aspects of these questions, because they incorporate basic rules of individual behaviour capturing the essence of the problem being explored. Global behaviour on these models are then accurate descriptions of their biological counterpart. So, by constructing simulations with very large number of parallel interacting agents, social biologists are now in better position to obtain relevant answers.

Mobile Cellular Automata (MCA) is a class of agent-based models very suited to study the emergence of collective behaviours out of individual interactions (Miramontes 1993). That is, to test whether a given global pattern may emerge from interindividual contacts among termite workers, a MCA model is build out of real-world parameters and compared to live termites. Convergence of results is interpreted as an evidence that the basic factors important to the production of the social behaviour have, in fact, been isolated (Cole and Cheshire 1996).

In this paper, we explore and compare the arising of coherent patterns of temporal activity in groups of termites and MCA of different sizes, arguing that such a convergence of results may undercover an universal property of social systems. Let us remember that MCA models are complex systems by their own right. The experiment was aimed to observe the essential traits of interacting behaviour among individuals that would lead to the emergence of social behaviour once the groups are sizable. As interactions between individuals increase in frequency, collective behaviour arises, and that is of course expected to be favoured by group size. Spontaneous activations should, therefore, tend to be dumped by induced activations in such a way that the number of spontaneous activations would decrease as the number of induced activations increases, both as a function of group size. Moreover, if collective behaviours are not a simple function of the superposition of the individual contributions (that is, collective patterns ‘emerge’ from the interindividual interactions), then decreasing spontaneous activations and increasing induced activations should obey a non-linear pattern.

We, therefore, expect to find a non-linear increase in the number of induced activations among individuals as group size increases, a non-linear decrease in the number of spontaneous interactions, a non-linear initial increase in the Kolmogorov-Shannon entropy and a subtle self-similar structure in the temporal dynamics of activity (Miramontes 1995; Solé et al. 1995).

## Materials and Methods

### Laboratory observations

Laboratory observations were made using *Cornitermes cumulans* (Kollar) (Isoptera: Termitidae) adult workers (third instar and beyond) collected in Viçosa state of Minas Gerais in Brazil. *Cornitermes* spp. are Neotropical termite species occurring in several habitats, including forests, “cerrados” (Brazilian savannas) and man-modified habitats, such as pastures or even gardens within cities, where they feed on living and dead grass and herbs (Cancello 1989). Several species of this genus, including *C. cumulans*, build large epigeous nests that are simultaneously inhabited by inquilines, such as other termite genera, ants, beetles, birds, snakes, etc (Redford 1984). Individuals were collected in the field from the inside of a nest. Portions of solid material from the nest were also brought together with the termites to the laboratory and placed in a plastic box measuring 30 x 40cm. This sample was stored in a controlled temperature biochamber for 24 hours at 25°C. The next day individuals were placed in sealed glass tubes measuring 9.5 x 14cm. Groups of different sizes (1, 2, 4, 6, 8, 16, 20 and 30 individuals) were created corresponding to different container densities (0.02, 0.04, 0.09, 0.14, 0.19, 0.38, 0.47 and 0.71, respectively). Each group was replicated four times. Tubes with termite groups were placed in a temperature controlled room (25°C) and allowed to acclimatise for three hours before the observations began. Two observers managed two replicates each. Light intensity in the room was the lowest possible to allow the work of the observers (250 Lx aprox). The number of active individuals was manually recorded each five minutes during ten continuous hours. In addition, observers recorded whether individuals pass from still to mobile and vice-versa spontaneously or under body-to-body contacts. Once these traits were noted, spontaneous and induced activations as a function of group size were estimated and compared with those obtained in computer models.

Spontaneous activations were estimated in termites after noting that individuals in large groups are less subject to isolation than those in smaller groups. Therefore when only one individual was recorded as active, it was assumed to be the product of a spontaneous activation.

Because of interactions, workers are able to induce the switching between tasks and to activate non-active individuals. Induced activations of non-active workers were estimated in three steps. First, all instances when only one individual was active was equated to zero, since it was assumed that those are likely the product of spontaneous activations. Then, the first-difference was calculated. Only positive quantities were considered since these represent activations rather than inactivations. Finally, all values were normalised.

The Kolmogorov-Shannon entropy is a complexity measure that gives information on the degree of order and the diversity of activity configurations in time. It also reveals the presence of order-disorder phase transitions. This quantity was evaluated following the classical Kolmogorov-Shannon entropy formalism:

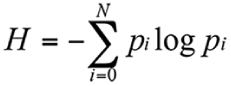

where *N* is the group size and *pi* is the probability of finding a number *i* of active termites, in a given time.

### Agent-based computer models

Agent-based computer models are now in widespread use for exploring a variety of biological phenomena including the study of behaviour in insects. They allow the modelling of any system of interacting objects sharing a set of minimum behavioural traits such as (1) agents react to interactions in order to modify their own internal states, (2) they would activate spontaneously if isolated from other agents, (3) would activate if touched directly by an already active agent and (4) would deactivate after a giving time. These traits of programmed behaviour are exactly the same observed in termites as discussed in the previous section, so that a qualitative comparison of their collective behaviour can be established.

The computer model is based on a formalism known as Mobile Cellular Automata, previously used for studying social behaviour in ants (Miramontes et al. 1993). Consider a 2-dimensional square lattice with *N* mobile automata moving as random walkers. The activity state *S_i_* of each object *a_i_* at time *t* is given by a function that couples the object's own activity with that of the others objects in the first lattice neighbourhood:

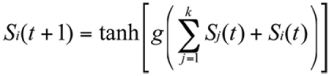

where *k* is the number of neighbours of *a_i_*. This state variable can take values in the interval [0,1]. When S_i_(*t*) is >0 then the individual may move, otherwise it remain motionless. Non-mobile automata may activate spontaneously with an activation probability pa or may activate after contact with an already active automaton. Two individuals cannot occupy the same cell simultaneously. If no free cell is in the first-neighborhood the individual remains in its current position until an empty cell becomes available. The parameter *g* is the gain of the *tanh* and it controls the degree of ‘excitability’ of the automaton. It is important to notice that *g* is a biological observable parameter and has been measured elsewhere in the context of ant interactions (Cole and Cheshire. 1996). The MCA model is very well-known for developing collective oscillatory behaviour as a function of the lattice density and for showing an order-disorder phase transition for a set of parameter values (Miramontes 1995; Solé et al. 1995). In the computer experiments the gain parameter *g* took the values 0.005 and 0.08 while the activation probability was set to 0.01. Lattice size was 10^2^.

**Figure 1.**
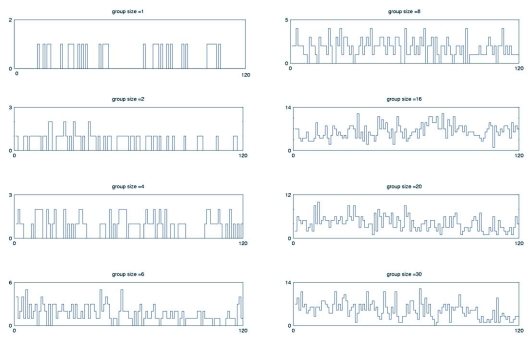
Example of temporal activity of groups of termites (*Cornitermes cumulans*) having different sizes. 120 observations were made each five minutes spanning to an interval often hours. Plots in each graph indicate the number of active individuals as a function of time, for selected samples. The observations were made in a conditioned observation room with dim light and controlled temperature.

## Results

The counting of active individuals gave a total of 28 time series (8 group sizes with 4 replicas) each containing 120 measures. Eight examples are shown in Figure 1 where it is noticeable that at high densities the activity per capita is larger on average than in individuals in lower densities. From data like these, the following measures were extracted: spontaneous activations, induced activations and entropy.

**Figure 2.**
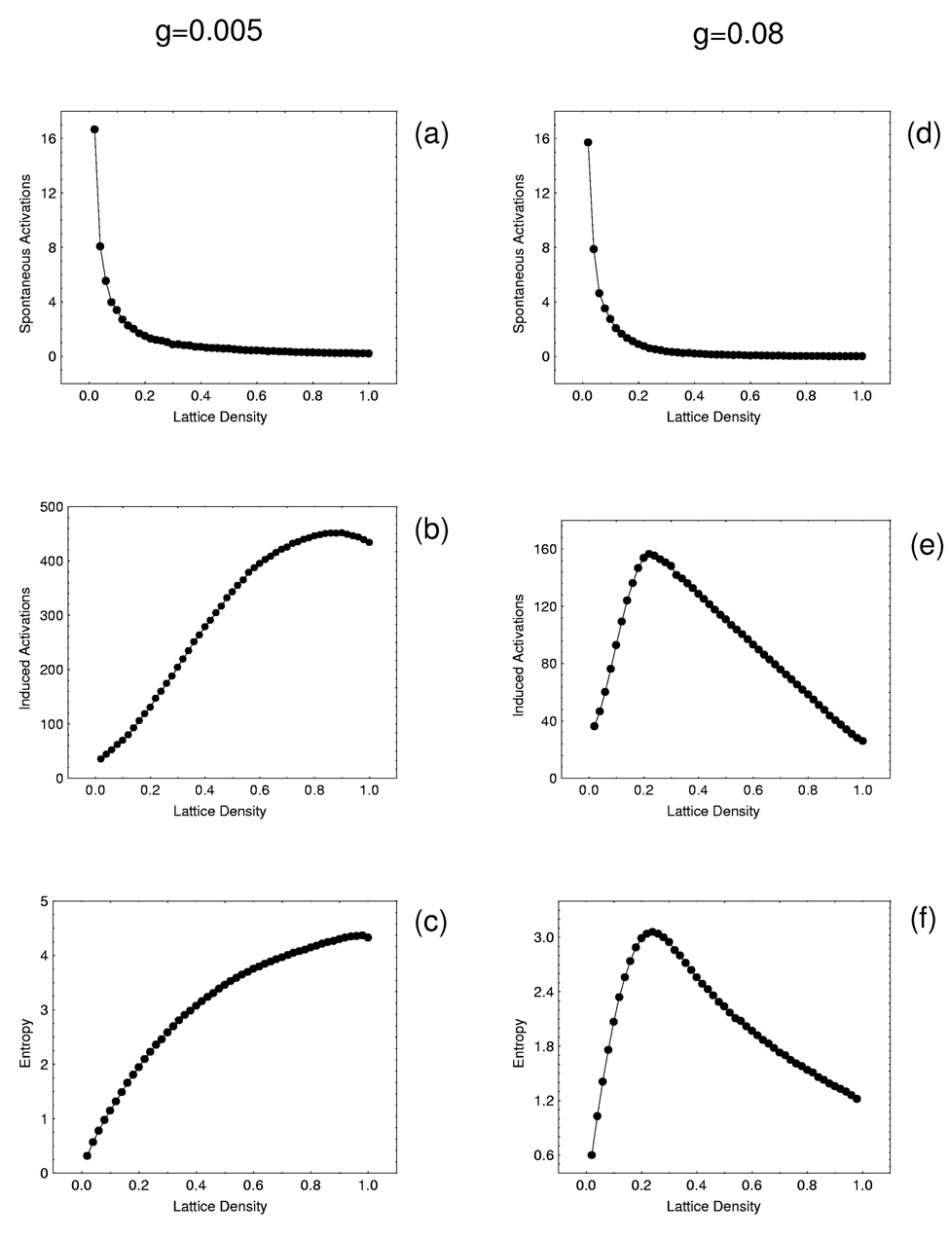
A Mobile Cellular Automata (MCA) model was used to qualitatively explore the behaviour of groups of automata under interactions. The model has been previously used in the study of ants and here it is used to explore complex dynamics in termites. Two sets of simulations were performed in order to measure, as a function of the group size or lattice density, the behaviour of spontaneous and induced activations and the KS-entropy. In (a) and (d), there is an exponential decay in the number of spontaneous activations measured for two different values of the gain parameter *g* (0.005 and 0.08 respectively). This behaviour is explained by the fact that, as the density is increased, less individuals are locally isolated and then are more engaged in social contacts. In (b) and (e), it is possible to observe the qualitative differences introduced when the gain parameter is modified. When the value is low, the induced activations grow logarithmically towards a maximum along the increase of the lattice density. In contrast (e) shows the case when *g* has a relatively large value. The impact of this is noticed by the maximum of induced activations occurring at a certain density value. Finally, in (c) and (d) the KS-entropy was measured and it is observed that a maximum may occur for a combination of values of *g.* This maximum is important because it signals the presence of a percolation-like phenomena separating a disordered from an ordered phase. In the first region the automata activates in an uncorrelated way, while in the second phase, temporal oscillations develop. No phase transition was detected in our experiment with *Cornitermes cumulons*. Lattice size = 10^2^. Activation probability = 0.01. each point is the average of 100 replicas.

## Spontaneous activations

**Figure 3.**
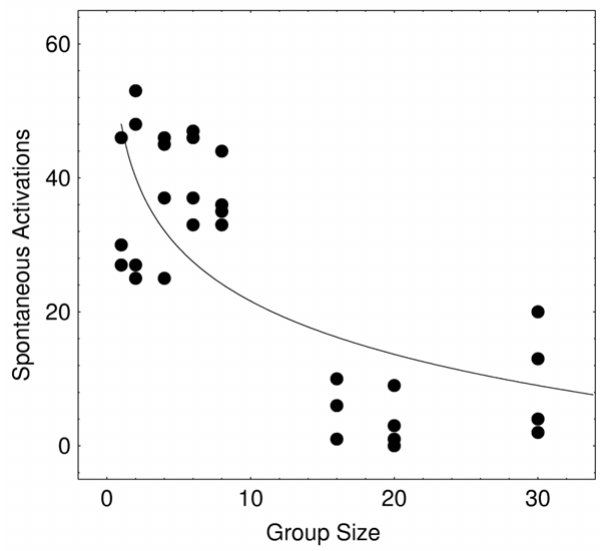
Non-linear decaying trend of the spontaneous activations as a function of the group size. This decaying is explained by the fact that, as the group size increases, the number of social contacts increases as well, then less individuals are locally isolated and less spontaneous activations are expected. The line is a guide to the eye only.

It was observed that isolated termites that are in resting states may spontaneously activate showing locomotion activity afterwards. These data were plotted against group size and a non-linear decaying trend was noticed (Figure 3). This decay process suggested that as the group size increased so did the level of interactions per capita and the whole group moved towards a more synchronised state. A decay in the number of spontaneous activations therefore revealed a coherent ordering of the collective behaviour. The foregoing reasoning is totally confirmed once it is contrasted with the agent-based computer model as shown in Figure 2. Here, the number of times a given agent goes from a zero-activity state to an active state was recorded revealing the same decaying trend as a function of group size.

## Induced activations

**Figure 4.**
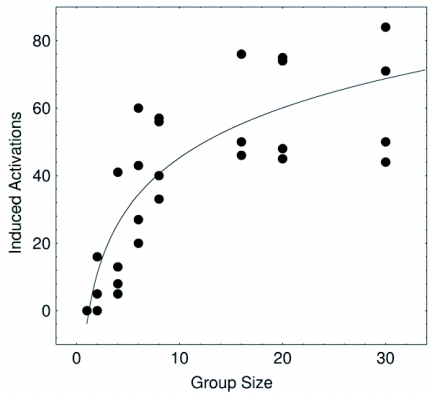
Induced activations increase along with the increase of the group size. This result is in agreement with the MCA model and is the result of a process of increasing social contacts. Notice the non-linear increasing pattern. The line is a guide to the eye only.

This measure is presented in Figure 4 where a non-linear increasing trend is evident. The results are in good qualitative agreement with those obtained with the MCA model when the value of the *g* parameter is not large, as in Figure 2(b).

## Order and entropy

**Figure 5.**
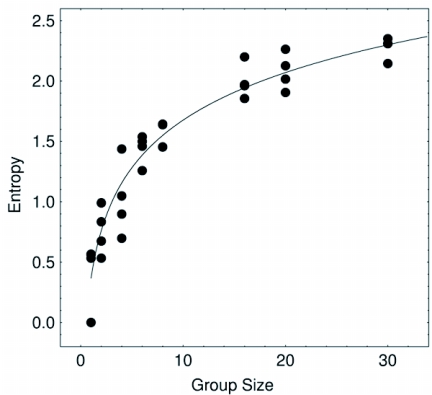
Entropy increase non-linearly along with the increase of the group size. This result is in agreement with the MCA model and is the result of a process of increasing social contacts. The line is a guide to the eye only.

Termite groups showed a non-linear trend to increase the entropy as the density increased. Since very high densities (»0.71) were not experimentally feasible, we do not know for sure if a peak was to be found further in the very high density region or if there was an asymptotic behaviour present without a peak of maximum value. Both states are theoretically possible and further experimental work is needed to solve this. The computer model gave a clue on this issue since the peak on the entropy is dependent of the gain parameter *g* as shown in Figure 5. Individuals with low values of this parameter (low response to interactions) do not show a peak in the entropy even for high density values. On the other hand, agents with a large value of the gain *g* do show a peak for low density values. Due to this, it is inconclusive whether an order-disorder transition was present in this experiment or if *C. cumulans* naturally operate in subcritical states below the density required for the order-disorder phase transition to manifest. This may be consistent with the fact that *C. cumulans* are not known to be highly excitable insects, at least as compared to other termites such as certain *Nasutitermes* spp or *Velocitermes* spp.

## Self-similar temporal structure of interactions

Patterns of social interactions may develop a subtle temporal non-trivial structure as collective coherence develops. A well-known technique for identifying these temporal structures is the so-called *chaos game* or Iterated Function Systems (IFS). This method allows the analysis of termite groups temporal activity in order to identify signatures of self-similar behaviour and is commonly used in the biological sciences and was used elsewhere in analysing insect dynamics (Miramontes et al. 1998; Miramontes et al. 2001; Hickel et al. 2003). IFS patterns as measured in termites seem to agree with the absence of white or Brownian noise.

## Discussion

Complex behaviour has been identified in social insects. It is now firmly established that isolated ant workers, for instance, may show temporal patterns of activations and locomotion characterised by deterministic chaos (Cole 1991a). While these patterns of disordered behaviour are present at the individual level, their complete colonies exhibit periodic cycles of ordered activity. Furthermore, it has been shown that as the group size of workers vary, its temporal dynamics varies accordingly, from disordered states into periodic synchronisation (Cole 1991b; Cole 1992). Using these behavioural traits, MCA computer models have helped to elucidate that such changes in the collective behaviour are the outcome of interactions alone in ants (Miramontes 1995; Miramontes & Rohani 2001; Solé et al. 1993) and in termites (Miramontes and DeSouza 1996), and that the transition from disorder into order is indeed what physicists call a phase transition characterised by critical fluctuations (1/*f* dynamics), where a number of information measures such as system entropy are maximised (Miramontes 1995). The phase transition has the density of the nest as its control parameter. Lately it has been shown that real ants self-organise to attain density values that seems to correspond to those in the range predicted by computer models. That would pose the nest as the verge of such a phase transition (Miramontes 1995; Solé et al. 1995)

In termites there is an effort to study how group size influences and initiate certain traits of collective behaviour, mainly by addressing the role of social facilitation in survival, poisoning resistance, disease transmission and reproduction (Miramonteset al.1996; DeSouza et al. 2001; DeSouza and Miramontes 2004; Santos et al. 2004; Muradian et al. 1999; Rosengaus al. 1998; Rosengaus al.2000; Pie el al.2004; Costa-Leonardo *et al*. 1999; Brent and Trainello 2001). Here we explored the interplay between group size and the temporal dynamics of spontaneous and induced activations, along with some complexity measures such as the group entropy and self-similarity in temporal patterns. In our laboratory experiment it was observed that the increase in group size translates into a non-linear decrease of the spontaneous activations, the same qualitative pattern that was observed in the computer simulations. The increase in group size had the effect of increasing the number of induced activations, the same pattern in the model and finally, the increase of the group size translated into an increase of entropy, both in the experiment and the model.

We did not try to parametrize the model, that is to evaluate precise values for the activation probaility *pa* or the gain porameter *g* for two reasons: first, it is known that the MCA model is quite roboust in its dynamic behaviour for a range of the values of these two parameters and, second, we are interested in a qualitative comparison of generic dynamical patterns between experiments, observations and models. While we acknowledge that evaluating exact parameter values may be of interest, it certainly goes beyond the scope of the present work.

**Figure 6.**
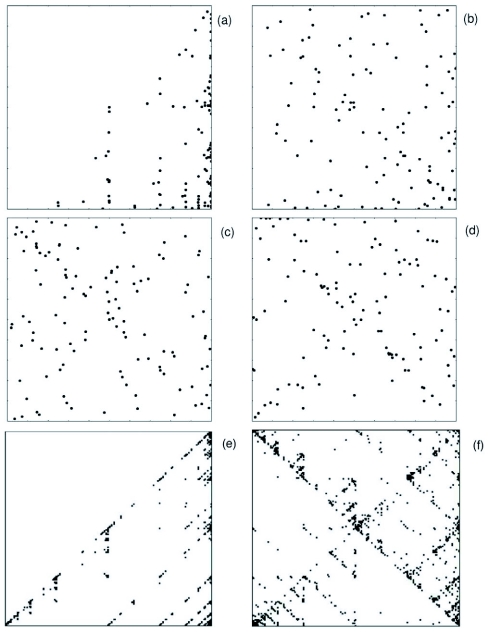
Iterated Function Analysis (IFS) of the dynamics of temporal activations. The IFS analysis works in the following way: the data set is sorted from the minimum to the maximum value and then subdivided into four segments such that each segment contains the same number of points (notice that the segments could be of different lengths). The original unsorted data set is then normalised and coarse-grained into four values, say 1,2,3 and 4, representing the quartile to where the data belong. The representation space is a square where the four corners are labelled 1,3,2,4 in a clockwise direction (starting in the lower left corner). Each value of the coarse-grained series is associated with the corner having the same number. A point is plotted half the way between the centre of the square and the first point of the series. A second point is plotted halfway between the first plotted point and the second point in the series, and so on. Results are shown of analysing the temporal patterns of group sizes of 8 (a), 16(b), 20(c) and 30(d). The time series are not large enough to form full-developed self-similar patterns in the IFS, nevertheless a comparison with MCA long series (e,f) shows that the patterns have similarities and so termite social activity have subtle temporal structure worth exploring in future research. MCA series were produced with 2000 long time-series. Density was low in (e) and high in (f).

Our results seem to point towards the existence of the same non-linear trends arising in other social insects. The use of agent-based models provide evidence that this may be the case. More detailed termite experiments are needed to establish this apparently universal property of social systems: non-linear trends in the emergence of sociality and the potential existence of order-disorder phase transitions with the group-size as the order parameter. It seems that our data is consistent with self-similar 1/*f* dynamics because the observed patterns are similar to those obtained with the MCA (Figure 6) and are also strikingly similar with those found in ants (Miramontes et al. 2001). However we do not conclude anything further because more data are needed. We hope that this work may serve as inspiration for a more complete exploration of this intriguing phenomenon.
